# The diagnostic value of serum gastrokine 1 (GKN1) protein in gastric cancer

**DOI:** 10.1002/cam4.2457

**Published:** 2019-08-03

**Authors:** Jung Hwan Yoon, Yong Gyu Park, Suk Woo Nam, Won Sang Park

**Affiliations:** ^1^ Department of Pathology, College of Medicine The Catholic University of Korea Seoul South Korea; ^2^ Functional RNomics Research Center, College of Medicine The Catholic University of Korea Seoul South Korea; ^3^ Department of Biostatistics, College of Medicine The Catholic University of Korea Seoul Republic of Korea

**Keywords:** biomarker, gastric cancer, GKN1, liquid biopsy

## Abstract

Early detection of cancer provides effective treatment and saves lives. The objective of this study was to determine whether serum gastrokine 1 (GKN1) protein is a gastric cancer‐specific diagnostic biomarker. The serum concentration of GKN1 in healthy individuals (median: 6.34 ng/μL, interquartile range (IQR): 5.66‐7.54 ng/μL) was significantly higher compared with the levels in gastric cancer patients (median: 3.48 ng/μL, IQR: 2.90‐4.11 ng/μL; *P* < .0001). At the optimum cutoff (4.94 ng/μL) of serum GKN1 protein, the sensitivity and specificity were 91.2% and 96.0%, respectively, for gastric cancer. Using serum GKN1 protein as the diagnostic reference, the ROC curve showed a satisfactory diagnostic efficacy with an AUC value of 0.9954 (95% CI 0.9919‐0.9988) and Youden index of 0.8740. In addition, the diagnostic accuracy of the serum GKN1 protein at the optimum cutoff was 0.9675. Interestingly, serum GKN1 concentrations in patients with advanced gastric cancer (AGC; median: 3.11 ng/μL, IQR: 2.72‐3.72 ng/μL) were lower than in patients with early gastric cancer (EGC; median: 4.31 ng/μL, IQR: 3.88‐4.88 ng/μL). The diagnostic accuracies at the optimum serum GKN1 cutoff were 0.8912 and 0.9589 for EGC and AGC, respectively. Furthermore, the serum GKN1 concentrations robustly discriminated the patients with gastric cancer from the patients with colorectal, liver, lung, breast, pancreatic, ovary, and prostatic cancers with AUC values greater than 0.94. These data suggest that serum GKN1 is a promising and highly specific diagnostic biomarker for the prompt detection of early and advanced gastric cancers.

## Graphical abstract

The serum GKN1 protein levels were lower in the gastric cancer patients compared to healthy individuals. The serum levels of GKN1 protein have clearly distinguished the gastric cancer patients from healthy individuals. The serum GKN1 protein could serve as an excellent diagnostic biomarker for early detection of gastric cancer.

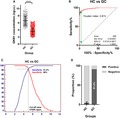

## 1 INTRODUCTION

Gastric cancer is the world's fourth most common cancer and the second leading cause of cancer death.^1^ Most patients with early gastric cancer (EGC) are asymptomatic, which often leads to late diagnosis and fatal outcomes.^2^ The diagnosis of gastric cancer relies on endoscopy and biopsy. Endoscopic and radiographic gastric cancer screening has been shown to increase the detection of gastric cancer, thus improving the survival rate.^3^, ^4^, ^5^ Despite important advances in the endoscopic screening and understanding of molecular carcinogenesis, and despite the low incidence of gastric cancer, the burden remains high. It is, therefore, imperative that early diagnostic biomarkers are developed to detect gastric cancer and predict the treatment outcome. Serum tumor markers, including carcinogenic embryonic antigen (CEA), and cancer antigen (CA) 125, have been used to detect malignancy and are currently used in the screening of certain cancers. However, cancer screening based on diagnostic serum biomarkers is not available for gastric cancer.

Human gastrokine 1 (GKN1) comprising 185 amino acid residues is a stomach‐specific protein which is produced by gastric mucus‐secreting cells; it is stored in cytoplasmic granules, and secreted as an exosomal protein.^6^, ^7^, ^8^ GKN1 plays an important role in maintaining mucosal integrity and homeostasis, and functions as a tumor suppressor by regulating cell proliferation and differentiation.^7^, ^9^, ^10^ Recently, we provided evidence suggesting that serum GKN1 protein is an informative diagnostic biomarker for gastric cancer.^7^ Here, serum GKN1 concentrations were measured in 200 healthy individuals and 1268 patients with cancer, and the clinical value of specific diagnostic marker for gastric cancer was analyzed. Finally, we report that the specificity and sensitivity of serum GKN1 concentration for the detection of gastric cancer were 91.2% and 96%, respectively.

## 2 METHODS

### 2.1 Ethical statement

We obtained written informed consent from all participants in accordance with the Declaration of Helsinki. The study was approved by the Institutional Review Board (IRB) of The Catholic University of Korea, College of Medicine (MC16SISI0132). No evidence of familial cancer was found in any of the patients.

### 2.2 Study population

The study included the sera obtained from 500 patients with gastric cancer, including 360 cases of advanced gastric cancer (AGC) and 140 with EGC. Based on the depth of invasion, gastric cancer patients were categorized into EGC and AGC. In addition, the sera from patients with defined hepatocellular carcinoma (HCC, n = 100), colorectal cancer (CRC, n = 100), non‐small cell carcinoma (NSCLC, 68 adenocarcinomas and 100 squamous cell carcinomas), invasive ductal carcinoma of breast (BRC, n = 200), pancreatic cancer (PAC, n = 100), ovarian cancer (OVC, n = 50), and prostatic cancer (PRC, n = 50) and healthy controls (n = 200) were obtained from five National Biobanks of Korea, Seoul Saint Mary Hospital, the Chonnam National University Hwasun Hospital, the Keimyung University Dongsan Medical Center, Ajou University Human Bio‐Resource Bank, and Inje University Busan Paik Hospital with IRB approval.

### 2.3 Measurement of serum GKN1 protein concentrations

We fractionated the whole blood samples obtained from 1268 cancer patients and 200 healthy subjects prior to operation. The sera from healthy and cancer subjects were immediately stored at −80°C after sampling. Samples were retrieved from storage and thawed at 4°C before the assay. To normalize for sample‐to‐sample variation, the total serum protein concentration was adjusted to 15 μg/mL with PBS. As GKN1 protein was present as an exosomal protein in human sera,^7^ we incubated the samples at 70°C for 10 min. Preoperative serum concentrations of GKN1 protein were determined using an ELISA kit (Cusabio, Wuhan, China), according to the manufacturer's instructions.

### 2.4 Defining GKN1 cutoff value in ROC analysis

To further evaluate the diagnostic value of the markers based on dichotomous classification, we conducted receiver‐operator characteristic (ROC) curve analysis. The ROC curve analysis was performed using the method introduced by Hanley and McNeil.^11^ The optimum cutoff value of GKN1 for the diagnosis of gastric cancers was defined in sera obtained from healthy individuals and patients with gastric cancer using the ROC curve and Youden index. Depending on the cutoff value of the serum GKN1 protein concentration, a range of sensitivity and specificity values was calculated. The serum GKN1 measurements in gastric cancer patients were used to determine the appropriate cutoff value of GKN1 for diagnosis of gastric cancer in the ethnic population studied. The sensitivity (true positive fraction, TPF), specificity (true negative fraction, TNF), false‐negative fraction (FNF), false‐positive fraction (FPF), positive predictive value (PPV), negative predictive value (NPV), accuracy, positive likelihood ratio (LR+), negative likelihood ratio (LR‐) and diagnostic odds ratio (DOR) for the cutoff value were calculated following the previously described method.^12^, ^13^, ^14^ We defined the cutoff value of serum GKN1 concentration by maximizing the overall prediction performance with the Youden's *J* index {*J*(_χ_) = (sensitivity_χ_ + specificity_χ_ − 1)} based on the ROC analysis.

### 2.5 Statistical analysis

We examined serum GKN1 protein concentrations in duplicate to verify the reproducibility of the findings. All statistical tests were performed using MedCalc (MedCalc Software, Mariakerke, Belgium), Graphpad prism (GraphPad Software, Inc, San Diego, CA), and SAS (SAS Institute; Cary, NC, USA). As serum GKN1 protein concentrations in healthy control subjects and in patients with gastric cancer showed markedly left‐skewed distributions in both group, we presented the results as medians and interquartile ranges (IQR, 25th and 75th percentiles), and compared the difference between groups by Mann‐Whitney *U*‐test. A *P*‐value less than .05 is considered as statistically significant.

## 3 RESULTS

### 3.1 Serum GKN1 is a specific biomarker for gastric cancer diagnosis

**Figure 1:**
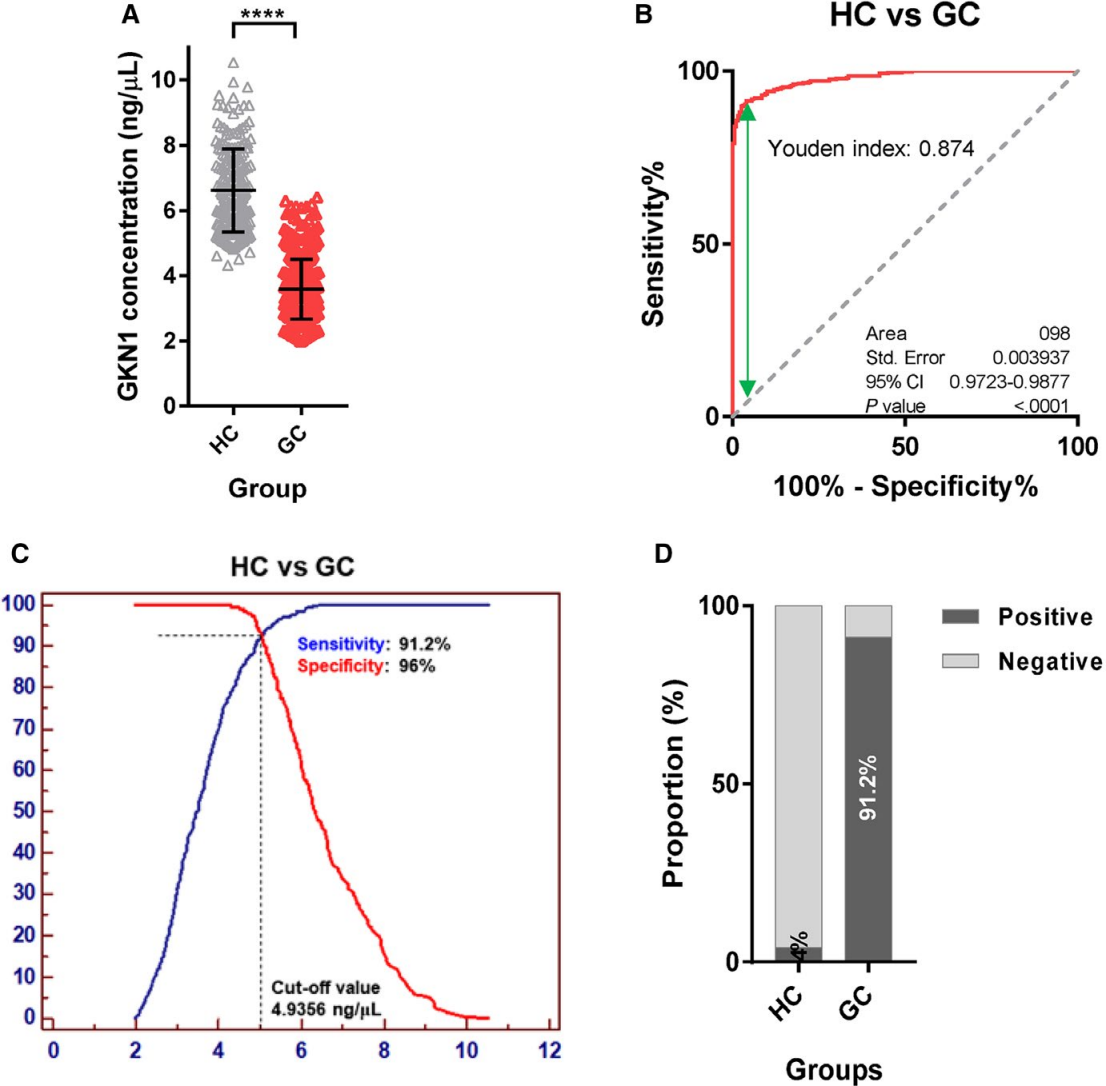
Serum GKN1 concentration in healthy control (HC) subjects and patients with gastric cancer (GC). (A) Scatter plot for serum GKN1 concentration in HC and in patients with GC (****, *P* < .0001). (B) ROC curve with Youden index for serum GKN1 in patients with GC vs HC. (C) Serum GKN1 protein at 4.94 ng/μL was considered as the optimal cutoff value for GC diagnosis using ROC curve analysis. (D) Rate of positive results at serum GKN1 cutoff value in HC and in patients with GC

To determine whether serum GKN1 protein concentration can be used as a novel biomarker for early detection of gastric cancer, we measured the serum GKN1 protein concentrations in 500 patients with gastric cancer and 200 healthy controls. As shown in Figure 1A and Table 1, the serum GKN1 protein concentrations were significantly lower in the gastric cancer patients (median: 3.48 ng/μL, IQR: 2.90‐4.11 ng/μL) compared with healthy individuals (median: 6.34 ng/μL, IQR: 5.66‐7.54 ng/μL; *P* < .0001). To evaluate the diagnostic potential of serum GKN1 concentration as an early detection biomarker in gastric cancer, the ROC curve analysis was performed. The serum GKN1 protein concentration clearly distinguished patients with gastric cancer from healthy individuals with an AUC value of 0.9954 and a Youden index of 0.8740 (Figure 1B). As shown in Figure 1C, the serum GKN1 concentration of 4.94 ng/μL was considered as the optimal cutoff value for gastric cancer diagnosis. At this cutoff value, the sensitivity and specificity for gastric cancer diagnosis were 91.2% and 96.0%, respectively (Figure 1C and Table 1). In addition, the PPV and NPV were 98.3% and 81.4%, respectively (Table 1). Moreover, the diagnostic accuracy and DOR at the serum GKN1 cutoff value were 0.9257 and 248.73, respectively (Table 1). Using this cutoff value for serum GKN1, 44 (8.8%) of the 500 patients with gastric cancer were negative for gastric cancer diagnosis (Figure 1D). Taken together, these results suggest that serum GKN1 protein represents a biomarker for gastric cancer diagnosis with exquisite sensitivity and specificity.

**Table 1:** Sensitivity, specificity, predictive value, accuracy, and diagnostic odds ratio for gastric cancers compared to healthy controls at cutoff value

	HC (n = 200)	GC (n = 500)
Age, year	56.1 ± 4.76	58.8 ± 13.9
GKN1, ng/μL		
Mean ± SD	6.62 ± 1.27	3.58 ± 0.92
Median	6.34	3.48
IQR	5.66‐7.54	2.90‐4.11
Cutoff, ng/μL		4.9356
TPF (sensitivity, %)		91.2
FNF (1‐sen, %)		8.8
TNF (specificity, %)		96
FPF (1‐spe, %)		4
PPV		0.982759
NPV		0.813559
LR+		22.8
LR‐		0.091667
Accuracy		0.925714
DOR		248.7273

Abbreviations: DOR, diagnostic odds ratio; FNF, false‐negative fraction; FPF, false‐positive fraction; GC, gastric cancer; HC, healthy control; IQR, interquartile range; LR, likelihood ratio; NPV, negative predictive value; PPV, positive predictive value; TNF, true‐negative fraction; TPF, true‐positive fraction.

### 3.2 Differentiation of healthy control subjects from early and advanced gastric cancer patients using serum GKN1 concentration

**Figure 2:**
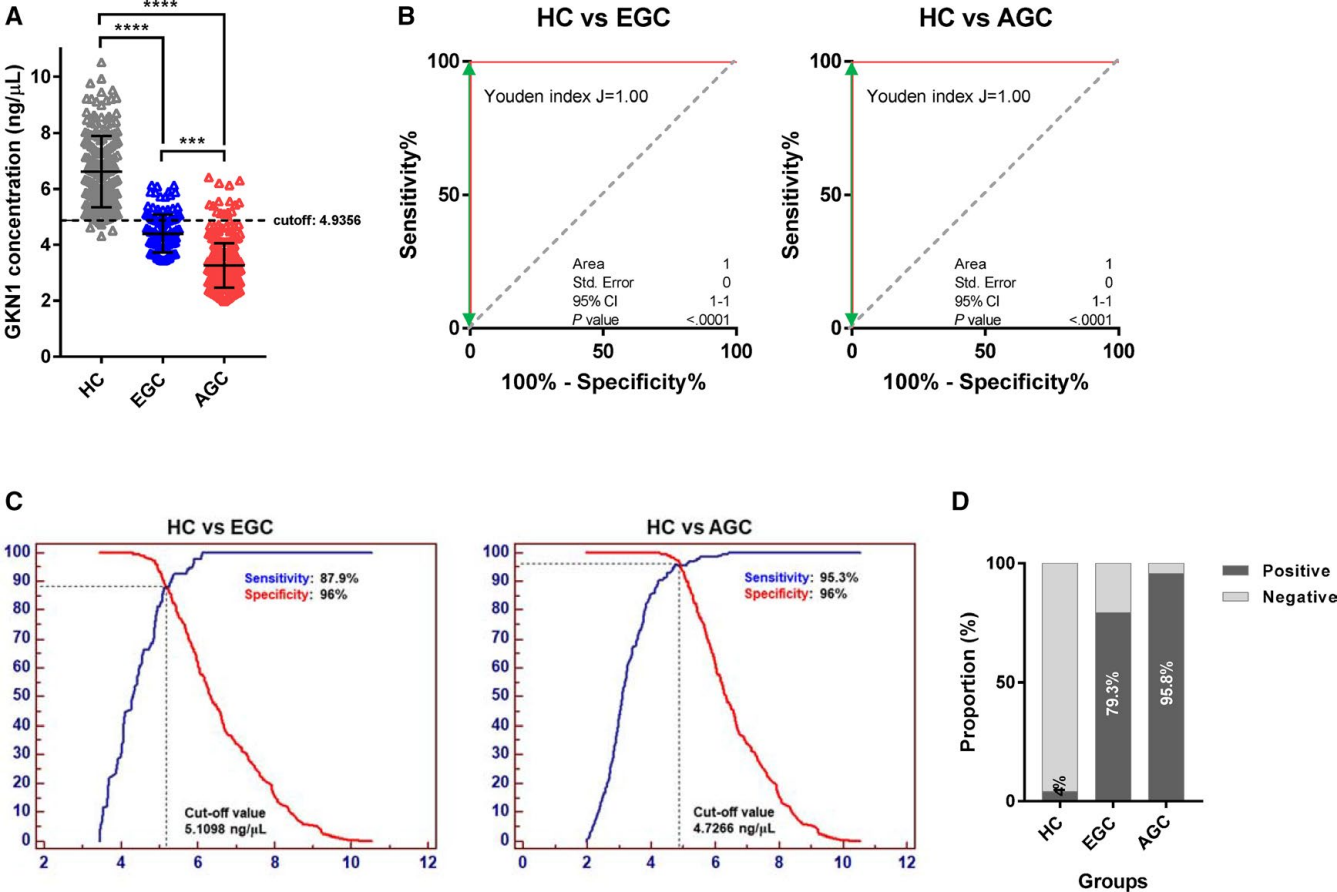
Serum GKN1 concentration in heathy control (HC) subjects and patients with early gastric cancers (EGC) and advanced gastric cancer (AGC). (A) Scatter plot of serum GKN1 protein concentration in HC and in patients with EGC and AGC. Dotted horizontal line represents the cutoff value of serum GKN1 concentration for GC diagnosis (***, *P* < .001 and ****, *P* < .0001). (B) ROC curve with Youden index for serum GKN1 for HC vs patients with EGC and AGC. (C) ROC curve analysis showed that serum GKN1 protein concentration at 5.11 ng/μL and 4.73 ng/μL represented the optimal cutoff values for EGC and AGC diagnosis, respectively. (D) Rate of positive results at a serum GKN1 cutoff value of 4.93 ng/μL in HC and in patients with EGC and AGC

Next, we investigated the diagnostic value of serum GKN1 concentrations in the detection of EGC and AGC. As expected, the serum GKN1 concentrations were significantly lower in both EGC (median: 4.31 ng/μL, IQR: 3.88‐4.88 ng/μL) and AGC (median: 3.11 ng/μL, IQR: 2.72‐3.72 ng/μL) than in healthy individuals (Figure 2A and Table 2). In particular, the serum GKN1 concentrations in patients with AGC were significantly lower than in patients with EGC (Figure 2A and Table 2). As expected, ROC curve analyses revealed that the serum GKN1 concentration clearly differentiated both EGC and AGC from healthy controls, with an AUC of 1.0000 (95% CI = 1.0000‐1.0000) and 1.0000 (95% CI = 1.0000‐1.0000), respectively, and with a Youden index of 1.00 and 1.00, respectively (Figure 2B). ROC curves showed that the optimum diagnostic cutoff values of serum GKN1 concentration were 5.11 ng/μL for EGC and 4.73 ng/μL for AGC, compared with healthy individuals (Figure 2C and Table 2). The cutoff value of 5.11 ng/μL in EGC yielded a sensitivity of 87.9% and a specificity of 96.0% for the diagnosis of EGC (Table 2). The cutoff value of 4.73 ng/μL yielded a diagnostic sensitivity of 95.3% and a diagnostic specificity of 96.0% for AGC (Table 2). When we selected 4.94 ng/μL as the cutoff value for serum GKN1 protein in both gastric cancers, the sensitivity and specificity were 79.3% and 96.0% for EGC, respectively, and 95.8% and 96.0% for AGC, respectively (Figure 2D and Table 3). The diagnostic accuracies at the serum GKN1 cutoff value were 0.8912 and 0.9589 for EGC and AGC, respectively (Table 3), suggesting that serum GKN1 is a potential diagnostic biomarker for both EGC and AGC.

**Table 2:** Sensitivity, specificity, predictive value, accuracy, and diagnostic odds ratio for early and advanced gastric cancers compared to healthy controls at cutoff values

	Normal (n = 200)	EGC (n = 140)	AGC (n = 360)
Age, year	56.1 ± 4.76	57.6 ± 8.3	59.1 ± 12.1
GKN1, μg/mL			
Mean ± SD	6.62 ± 1.27	4.4 ± 0.68	3.26 ± 0.8
Median	6.34	4.31	3.11
IQR	5.66‐7.54	3.88‐4.88	2.72‐3.72
Cutoff, μg/mL		5.1098	4.7266
TPF (sensitivity, %)		87.9	95.3
FNF (1‐sen, %)		12.1	4.7
TNF (specificity, %)		96	96
FPF (1‐spe, %)		4	4
PPV		0.938931	0.977208
NPV		0.91866	0.91866
LR+		21.96429	23.81944
LR‐		0.126488	0.04919
Accuracy		0.926471	0.955357
DOR		173.6471	484.2353

Abbreviations: AGC, advanced gastric cancer; DOR, diagnostic odds ratio; EGC, early gastric cancer; FNF, false‐negative fraction; FPF, false‐positive fraction; HC, healthy control; IQR, interquartile range; LR, likelihood ratio; NPV, negative predictive value; PPV, positive predictive value; TNF, true‐negative fraction; TPF, true‐positive fraction.

**Table 3:** Sensitivity, specificity, predictive value, accuracy, and diagnostic odds ratio for early and advanced gastric cancers compared to healthy controls at cutoff value for gastric cancers

	Normal (n = 200)	EGC (n = 140)	AGC (n = 360)
Age, year	56.1 ± 4.76	57.6 ± 8.3	59.1 ± 12.1
GKN1, ng/μL			
Mean ± SD	6.62 ± 1.27	4.4 ± 0.68	3.26 ± 0.8
Median	6.34	4.31	3.11
IQR	5.66‐7.54	3.88‐4.88	2.72‐3.72
Cutoff, ng/μL		4.9356	4.9356
TPF (sensitivity, %)		79.3	95.8
FNF (1‐sen, %)		20.7	4.2
TNF (specificity, %)		96	96
FPF (1‐spe, %)		4	4
PPV		0.932773	0.977337
NPV		0.868778	0.927536
LR+		19.82143	23.95833
LR‐		0.215774	0.043403
Accuracy		0.891176	0.958929
DOR		91.86207	552

Abbreviations: AGC, advanced gastric cancer; DOR, diagnostic odds ratio; EGC, early gastric cancer; FNF, false‐negative fraction; FPF, false‐positive fraction; HC, healthy control; IQR, interquartile range; LR, likelihood ratio; NPV, negative predictive value; PPV, positive predictive value; TNF, true‐negative fraction; TPF, true‐positive fraction.

### 3.3 Differentiation of gastric cancer patients from non‐gastric cancer patients using serum GKN1 concentration

**Figure 3:**
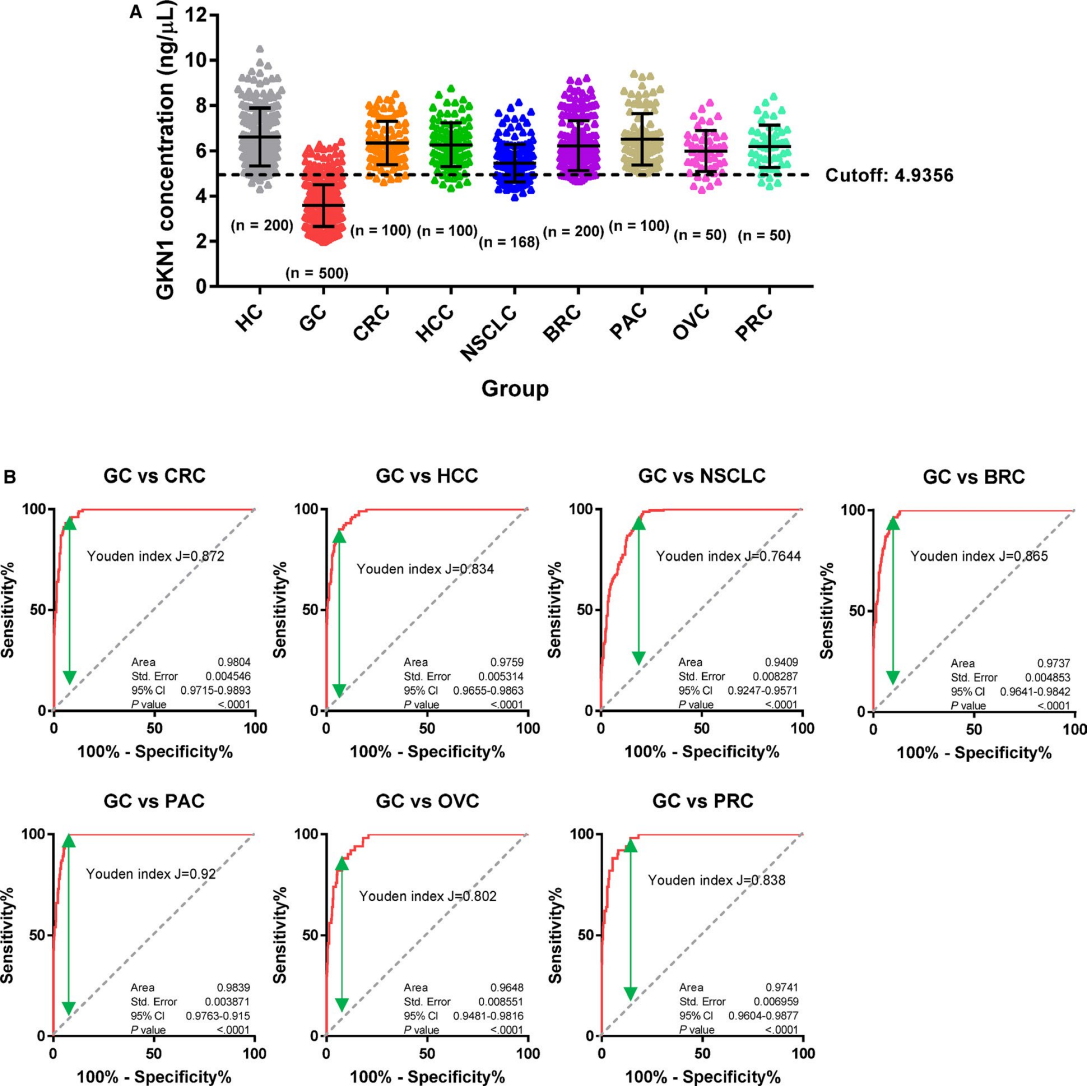
Serum GKN1 concentration in healthy control (HC) subjects and patients with colorectal cancer (CRC), hepatocellular carcinoma (HCC), non‐small cell lung cancer (NSCLC), breast cancer (BRC), pancreatic cancer (PAC), ovary cancer (OVC), and prostate cancer (PRC). (A) Scatter plot for serum GKN1 protein concentration in HC and in patients with GC, CRC, HCC, NSCLC, BRC, PAC, OVC, and PRC. Dotted horizontal line shows a cutoff value of serum GKN1 concentration for GC diagnosis. (B) ROC curve with Youden index for serum GKN1 in patients with GC versus patients with CRC, HCC, NSCLC, BRC, PAC, OVC, and PRC

Next, we investigated whether serum GKN1 protein concentration discriminated healthy individuals from patients with non‐gastric cancers including HCC, CRC, NSCLC, BRC, PAC, OVC, and PRC. The serum GKN1 concentrations of healthy individuals and patients with non‐gastric cancers are presented in Table S1. Notably, the serum GKN1 concentrations in patients with HCC, CRC, NSCLC, BRC, PAC, OVC, and PRC did not differ from those in normal healthy sera (Figure 3A and Table S1). In addition, the AUC values for the diagnosis of other cancers based on serum GKN1 protein showed a dissatisfactory diagnostic efficacy (data not shown). When we compared serum GKN1 concentrations between patients with gastric cancer and patients with non‐gastric cancers, serum GKN1 concentration distinguished patients with gastric cancer from those with the foregoing non‐gastric cancers with AUC values of >0.94 (Figure 3B) and diagnostic accuracies of >0.86 (Table S2), suggesting that serum GKN1 protein may be a gastric cancer‐specific diagnostic biomarker.

## 4 DISCUSSION

Recent advances in proteomics have provided novel diagnostic techniques, such as liquid biopsy, for the detection of cancer, but many protein‐based tests have not reached the sensitivity and specificity needed for clinical applications.^15^ Blood‐based proteins including exosomal proteins provide direct information during tumor development and progression in “real time”.^16^ In particular, exosomal proteins are highly stable and have a higher sensitivity than proteins directly detected in blood.^16^ GKN1 is specifically expressed in normal gastric mucosal epithelial cells and plays an important role in maintaining the integrity and homeostasis of gastric mucosa.^6^ We and others have reported that GKN1 expression was reduced in gastric mucosa infected with *Helicobacter pylori* and was significantly downregulated in gastric cancer tissues.^17^, ^18^ In a previous work, we found that GKN1 is secreted as one of the exosomal cargo proteins and serum GKN1 protein is an informative diagnostic biomarker for gastric cancer.^7^ Because serum is an invaluable disease biomarker reservoir and serum proteins act as biomarkers of various disease status including cancers, we examined the potential of serum GKN1 protein concentration as a gastric cancer‐specific diagnostic biomarker. Given its high sensitivity and specificity, serum GKN1 protein can be used as a serological marker for the diagnosis of gastric cancer. Interestingly, the serum concentrations of GKN1 protein were significantly more reduced in patients with gastric cancer than in normal healthy individuals and clearly distinguished the patients, with an AUC value of 0.98, which represents a perfect value for clinical application. At a serum GKN1 cutoff value of 4.94 ng/μL the sensitivity, specificity, Youden index, diagnostic accuracy and DOR for gastric cancer diagnosis were 91.2%, 96%, 0.8740, 0.9257, and 248.73, respectively. Thus, these results suggest that serum GKN1 protein serves as an excellent diagnostic biomarker to distinguish patients with gastric cancer from normal healthy individuals.

EGC is defined as gastric carcinoma confined to the mucosa and/or submucosa (T1), irrespective of lymph node involvement, according to the Japanese Classification of Gastric Carcinoma.^19^ When the tumor invades the muscularis propria (T2), it is classified as AGC. In the present study, the serum GKN1 protein concentrations in patients with AGC were significantly lower than in patients with EGC. In addition, the ROC curve can be used to distinguish patients with EGC from those with AGC based on an AUC value of 0.870, which represents a good diagnostic value. Interestingly, Myc plays a central role in cell growth and proliferation, metabolism, differentiation, apoptosis, and angiogenesis.^20^ Myc heteromerized with Max to acquire DNA‐binding activity and Myc/Max dimers bind to the promoters of specific target genes.^21^ Previously, we found that Myc and Max proteins in exosomes derived from gastric cancer cells downregulated the expression and promoter binding activity of NKX6.3, which is a transcription factor for GKN1.^7^ The amplification and overexpression of Myc are frequently detected in gastric cancers.^22^, ^23^, ^24^, ^25^, ^26^ Importantly, the expression of Myc was higher in advanced GC than in early stage GC^27^, ^28^ and in metastasis.^29^ Therefore, it is likely that the overexpression of Myc‐derived from gastric cancer cells may downregulate the expression and secretion of exosomal GKN1 protein in nonneoplastic gastric mucosal epithelial cells. Further studies are strongly recommended to identify the molecular mechanism underlying low serum GKN1 protein concentrations in gastric cancer patients.

Gastroscopic examination is the most reliable method for the diagnosis of gastric cancer. However, upper endoscopy is an uncomfortable procedure, and incurs risks of intubation and sedation, infection, false‐negative, false‐positive results, and overdiagnosis.^30^ In addition, upper endoscopy is expensive and needs technological expertise. Notably, the lack of symptoms or signs in the early stages of gastric cancer prevents prompt detection and treatment. Because detection of serum tumor markers is inexpensive and convenient, novel blood‐borne biomarkers that can detect gastric cancer early can be used in sensitive and specific screening programs. Extensive studies have demonstrated several serological biomarkers for gastric cancer, including CEA, CA 19‐9, and CA 72‐4. However, these serum biomarkers are not satisfactory for gastric cancer diagnosis, due to their low sensitivity and specificity.^31^, ^32^, ^33^ To overcome these limitations, we suggest serum GKN1 protein as a novel gastric cancer‐specific diagnostic biomarker that distinguishes patients with gastric cancer from healthy controls. Analysis of preoperative and periodic postoperative serum GKN1 protein concentrations can be used to predict the response to treatment and monitor the recurrence of gastric cancer.

Here, serum GKN1 concentrations were examined in patients with HCC, CRC, NSCLC, BRC, PAC, OVC, and PRC to further confirm the potential diagnostic utility of serum GKN1 concentrations in differentiating healthy individuals from patients with non‐gastric cancers. No significant difference was found in the serum GKN1 concentrations between healthy controls and patients with non‐gastric cancers. The ROC curve showed unsatisfactory diagnostic efficacy. However, serum GKN1 concentration discriminated patients with gastric cancer from those with non‐gastric cancers. Thus, we conclude that serum GKN1 protein could be a gastric cancer‐specific diagnostic biomarker.

The present study had specific limitations. First, the sample size was relatively small and the analysis was performed in the Korean population. The application of serum GKN1 concentration as a diagnostic marker for gastric cancer worldwide requires a multinational study. In addition, further studies are strongly needed to evaluate whether the cutoff can be used for the detection of gastric cancer. Second, this was a retrospective study. Clinical application requires prospective studies to evaluate the diagnostic potential of serum GKN concentration in gastric cancer.

In conclusion, serum concentrations of GKN1 clearly distinguished patients with gastric cancer from healthy controls and those with non‐gastric cancers. To investigate the role of serum GKN1 as a useful surveillance biomarker for the assessment of the therapeutic response in gastric cancer patients, a long‐term follow‐up of gastric cancer patients undergoing surgery is needed. Although clinical trials for the screening of serum GKN1 protein concentration prior to clinical application are strongly required, the key strength of this study is that serum GKN1 protein can be used as a potential serological marker for early detection of gastric cancer.

## CONFLICT OF INTEREST

The authors disclose no potential conflict of interest.

## AUTHOR CONTRIBUTIONS

WSP and JHY conceptualized and designed the study. WSP financially supported. JHY collected and assembled the data. WSP and JHY performed data analysis and interpretation. YGP performed statistical analysis. JHY, SWN and WSP involved in manuscript writing and gave the final approval of manuscript and accountable for all aspects of the work.

## References

[1] Van Cutsem E , Sagaert X , Topal B , Haustermans K , Prenen H . Gastric cancer. Lancet. 2016;388:2654‐2664. 27156933 10.1016/S0140-6736(16)30354-3

[2] Sobota RS , Kodaman N , Mera R , et al. Epigenetic and genetic variation in GATA5 is associated with gastric disease risk. Hum Genet. 2016;135:895‐906. 27225266 10.1007/s00439-016-1687-1 PMC4947561

[3] Hamashima C , Shabana M , Okada K , Okamoto M , Osaki Y . Mortality reduction from gastric cancer by endoscopic and radiographic screening. Cancer Sci. 2015;106:1744‐1749. 26432528 10.1111/cas.12829 PMC4714659

[4] Jun JK , Choi KS , Lee H‐Y , et al. Effectiveness of the Korean National Cancer screening program in reducing gastric cancer mortality. Gastroenterology. 2017;152:1319‐1328 e1317. 28147224 10.1053/j.gastro.2017.01.029

[5] Khanderia E , Markar SR , Acharya A , Kim Y , Kim YW , Hanna GB . The influence of gastric cancer screening on the stage at diagnosis and survival: a meta‐analysis of comparative studies in the far East. J Clin Gastroenterol. 2016;50:190‐197. 26844858 10.1097/MCG.0000000000000466

[6] Martin TE , Powell CT , Wang Z , et al. A novel mitogenic protein that is highly expressed in cells of the gastric antrum mucosa. Am J Physiol Gastrointest Liver Physiol. 2003;285:G332‐G343. 12851218 10.1152/ajpgi.00453.2002

[7] Yoon JH , Ham I‐H , Kim O , et al. Gastrokine 1 protein is a potential theragnostic target for gastric cancer. Gastric Cancer. 2018;21:956‐967. 29704153 10.1007/s10120-018-0828-8

[8] Yoshikawa Y , Mukai H , Hino F , Asada K , Kato I . Isolation of two novel genes, down‐regulated in gastric cancer. Jpn J Cancer Res. 2000;91:459‐463. 10835488 10.1111/j.1349-7006.2000.tb00967.x PMC5926377

[9] Yoon JH , Choi WS , Kim O , Park WS . The role of gastrokine 1 in gastric cancer. J Gastric Cancer. 2014;14:147‐155. 25328759 10.5230/jgc.2014.14.3.147 PMC4199881

[10] Yoon JH , Choi YJ , Choi WS , et al. GKN1‐miR‐185‐DNMT1 axis suppresses gastric carcinogenesis through regulation of epigenetic alteration and cell cycle. Clin Cancer Res. 2013;19:4599‐4610. 23846337 10.1158/1078-0432.CCR-12-3675

[11] Hanley JA , McNeil BJ . A method of comparing the areas under receiver operating characteristic curves derived from the same cases. Radiology. 1983;148:839‐843. 6878708 10.1148/radiology.148.3.6878708

[12] Fedarko NS , Jain A , Karadag A , Van Eman MR , Fisher LW . Elevated serum bone sialoprotein and osteopontin in colon, breast, prostate, and lung cancer. Clin Cancer Res. 2001;7:4060‐4066. 11751502

[13] Florkowski CM . Sensitivity, specificity, receiver‐operating characteristic (ROC) curves and likelihood ratios: communicating the performance of diagnostic tests. Clin Biochem Rev. 2008;29(Suppl 1):S83‐S87. 18852864 PMC2556590

[14] Sonego P , Kocsor A , Pongor S . ROC analysis: applications to the classification of biological sequences and 3D structures. Brief Bioinform. 2008;9:198‐209. 18192302 10.1093/bib/bbm064

[15] Heitzer E , Perakis S , Geigl JB , Speicher MR . The potential of liquid biopsies for the early detection of cancer. NPJ Precis Oncol. 2017;1:36. 29872715 10.1038/s41698-017-0039-5 PMC5871864

[16] Li A , Zhang T , Zheng M , Liu Y , Chen Z . Exosomal proteins as potential markers of tumor diagnosis. J Hematol Oncol. 2017;10:175. 29282096 10.1186/s13045-017-0542-8 PMC5745959

[17] Nardone G , Martin G , Rocco A , et al. Molecular expression of Gastrokine 1 in normal mucosa and in Helicobacter pylori‐related preneoplastic and neoplastic gastric lesions. Cancer Biol Ther. 2008;7:1890‐1895. 18927498 10.4161/cbt.7.12.6936

[18] Yoon JH , Song JH , Zhang C , et al. Inactivation of the Gastrokine 1 gene in gastric adenomas and carcinomas. J Pathol. 2011;223:618‐625. 21341273 10.1002/path.2838

[19] Japanese Gastric Cancer Association . Japanese Classification of Gastric Carcinoma ‐ 2nd English Edition. Gastric Cancer. 1998;1:10‐24. 11957040 10.1007/s101209800016

[20] Gabay M , Li Y , Felsher DW . MYC activation is a hallmark of cancer initiation and maintenance. Cold Spring Harb Perspect Med. 2014;4:a014241. 10.1101/cshperspect.a014241 PMC4031954 24890832

[21] Milne AN , Sitarz R , Carvalho R , Carneiro F , Offerhaus GJ . Early onset gastric cancer: on the road to unraveling gastric carcinogenesis. Curr Mol Med. 2007;7:15‐28. 17311530 10.2174/156652407779940503

[22] Calcagno DQ , Freitas VM , Leal MF , et al. MYC, FBXW7 and TP53 copy number variation and expression in gastric cancer. BMC Gastroenterol. 2013;13:141. 24053468 10.1186/1471-230X-13-141 PMC3851138

[23] Calcagno DQ , Guimaraes AC , Leal MF , et al. MYC insertions in diffuse‐type gastric adenocarcinoma. Anticancer Res. 2009;29:2479‐2483. 19596917

[24] Calcagno DQ , Leal MF , Seabra AD , et al. Interrelationship between chromosome 8 aneuploidy, C‐MYC amplification and increased expression in individuals from northern Brazil with gastric adenocarcinoma. World J Gastroenterol. 2006;12:6207‐6211. 17036397 10.3748/wjg.v12.i38.6207 PMC4088119

[25] Cowling VH , Cole MD . Mechanism of transcriptional activation by the Myc oncoproteins. Semin Cancer Biol. 2006;16:242‐252. 16935524 10.1016/j.semcancer.2006.08.001

[26] Onoda N , Maeda K , Chung YS , et al. Overexpression of c‐myc messenger RNA in primary and metastatic lesions of carcinoma of the stomach. J Am Coll Surg. 1996;182:55‐59. 8542090

[27] Xu AG , Li SG , Liu JH , Gan AH . Function of apoptosis and expression of the proteins Bcl‐2, p53 and C‐myc in the development of gastric cancer. World J Gastroenterol. 2001;7:403‐406. 11819799 10.3748/wjg.v7.i3.403 PMC4688731

[28] Zhang GX , Gu YH , Zhao ZQ , et al. Coordinate increase of telomerase activity and c‐Myc expression in Helicobacter pylori‐associated gastric diseases. World J Gastroenterol. 2004;10:1759‐1762. 15188501 10.3748/wjg.v10.i12.1759 PMC4572264

[29] Kozma L , Kiss I , Hajdu J , Szentkereszty Z , Szakall S , Ember I . C‐myc amplification and cluster analysis in human gastric carcinoma. Anticancer Res. 2001;21:707‐710. 11299830

[30] Hamashima C . Overdiagnosis of gastric cancer by endoscopic screening. World J Gastrointest Endosc. 2017;9:55‐60. 28250897 10.4253/wjge.v9.i2.55 PMC5311473

[31] Gaspar MJ , Arribas I , Coca MC , Diez‐Alonso M . Prognostic value of carcinoembryonic antigen, CA 19–9 and CA 72–4 in gastric carcinoma. Tumour Biol. 2001;22:318‐322. 11553862 10.1159/000050633

[32] Ishigami S , Natsugoe S , Hokita S , et al. Clinical importance of preoperative carcinoembryonic antigen and carbohydrate antigen 19–9 levels in gastric cancer. J Clin Gastroenterol. 2001;32:41‐44. 11154168 10.1097/00004836-200101000-00010

[33] Reiter W , Stieber P , Reuter C , et al. Prognostic value of preoperative serum levels of CEA, CA 19–9 and CA 72–4 in gastric carcinoma. Anticancer Res. 1997;17:2903‐2906. 9329559

