# Supplementary material for: The diagnostic value of serum gastrokine 1 (GKN1) protein in gastric cancer
Source: Cancer Med. 2019 Aug 3;8(12):5507–14. doi: 10.1002/cam4.2457 (PMC6745860; doi:10.1002/cam4.2457)
Supplement: Supplementary file 1 [file CAM4-8-5507-s001.docx]

**Supplementary Table 1.** Sensitivity, specificity, predictive value, accuracy, and diagnostic odds ratio for each cancer compared to healthy controls at cut-off values for gastric cancers.

|  | HC | CRC | HCC | NSCLC | BRC | PAC | OVC | PRC |
| --- | --- | --- | --- | --- | --- | --- | --- | --- |
|  | (n=200) | (n=100) | (n=100) | (n=168) | (n=200) | (n=100) | (n=50) | (n=50) |
| Age, yr | 56.1 ± 4.76 | 63.4 ± 14.6 | 60.5 ± 12.5 | 66.7 ± 9.6 | 51.2 ± 10.8 | 65 ± 9.5 | 58.3 ± 10.2 | 69.3 ± 5.1 |
| GKN1, ng/μL | 6.62 ± 1.27 | 6.36 ± 0.95 | 6.28 ± 0.96 | 5.6 ± 0.87 | 6.23 ± 1.1 | 6.52 ± 1.13 | 6 ± 0.9 | 6.2 ± 0.93 |
| TPF (sensitivity, %) |  | 4 | 7 | 24.4 | 3.5 | 0 | 12 | 86 |
| FNF (1-sen, %) |  | 96 | 93 | 75.6 | 96.5 | 100 | 88 | 14 |
| TNF (specificity, %) |  | 97.5 | 97.5 | 97.5 | 97.5 | 97.5 | 97.5 | 97.5 |
| FPF (1-spe, %) |  | 2.5 | 2.5 | 2.5 | 2.5 | 2.5 | 2.5 | 2.5 |
| PPV |  | 0.444444 | 0.583333 | 0.891304 | 0.583333 | 0 | 0.545455 | 0.244318 |
| NPV |  | 0.670103 | 0.677083 | 0.60559 | 0.502577 | 0.661017 | 0.8159 | 0.905405 |
| LR+ |  | 1.6 | 2.8 | 9.761905 | 1.4 | 0 | 4.8 | 1.293233 |
| LR- |  | 0.984615 | 0.953846 | 0.775336 | 0.989744 | 1.025641 | 0.902564 | 0.41791 |
| Accuracy |  | 0.663333 | 0.673333 | 0.641304 | 0.505 | 0.65 | 0.804 | 0.796 |
| DOR |  | 1.625 | 2.935484 | 12.59055 | 1.414508 | 0 | 5.318182 | 3.094522 |

HC, healthy control; CRC, colorectal cancer; HCC, hepatocellular carcinoma; NSCLC, non-small cell lung cancer; BRC, invasive ductal carcinoma of breast; PAC, pancreatic cancer; OVC, ovarian cancer; PRC, prostatic cancer; TPF, true positive fraction; FNF, false negative fraction; TNF, true negative fraction; FPF, false positive fraction; PPV, positive predictive value; NPV, negative predictive value; LR, likelihood ratio; DOR, diagnostic odds ratio
