# Supplementary material for: The diagnostic value of serum gastrokine 1 (GKN1) protein in gastric cancer
Source: Cancer Med. 2019 Aug 3;8(12):5507–14. doi: 10.1002/cam4.2457 (PMC6745860; doi:10.1002/cam4.2457)
Supplement: Supplementary file 2 [file CAM4-8-5507-s002.docx]

**Supplementary Table 2.** Sensitivity, specificity, predictive value, accuracy, and diagnostic odds ratio for each cancer compared to gastric cancers at cut-off values for gastric cancers.

|  | GC | CRC | HCC | NSCLC | BRC | PAC | OVC | PRC |
| --- | --- | --- | --- | --- | --- | --- | --- | --- |
|  | (n=500) | (n=100) | (n=100) | (n=168) | (n=200) | (n=100) | (n=50) | (n=50) |
| Age, yr | 58.8 ± 13.9 | 63.4 ± 14.6 | 60.5 ± 12.5 | 66.7 ± 9.6 | 51.2 ± 10.8 | 65 ± 9.5 | 58.3 ± 10.2 | 69.3 ± 5.1 |
| GKN1, ng/μL | 3.58 ± 0.92 | 6.36 ± 0.95 | 6.28 ± 0.96 | 5.6 ± 0.87 | 6.23 ± 1.1 | 6.52 ± 1.13 | 6 ± 0.9 | 6.2 ± 0.93 |
| TPF (sensitivity, %) |  | 91.2 | 91.2 | 91.2 | 91.2 | 91.2 | 91.2 | 91.2 |
| FNF (1-sen, %) |  | 8.8 | 8.8 | 8.8 | 8.8 | 8.8 | 8.8 | 8.8 |
| TNF (specificity, %) |  | 95 | 92 | 72.6 | 93.5 | 99 | 88 | 92 |
| FPF (1-spe, %) |  | 5 | 8 | 27.4 | 6.5 | 1 | 12 | 8 |
| PPV |  | 0.989154 | 0.982759 | 0.908367 | 0.972281 | 0.997812 | 0.987013 | 0.991304 |
| NPV |  | 0.683453 | 0.676471 | 0.73494 | 0.809524 | 0.692308 | 0.5 | 0.511111 |
| LR+ |  | 18.24 | 11.4 | 3.330783 | 14.03077 | 91.2 | 7.6 | 11.4 |
| LR- |  | 0.092632 | 0.095652 | 0.12118 | 0.094118 | 0.088889 | 0.1 | 0.095652 |
| Accuracy |  | 0.918333 | 0.913333 | 0.865269 | 0.918571 | 0.925 | 0.909091 | 0.912727 |
| DOR |  | 196.9091 | 119.1818 | 27.48617 | 149.0769 | 1026 | 76 | 119.1818 |

GC, gastric cancer; CRC, colorectal cancer; HCC, hepatocellular carcinoma; NSCLC, non-small cell lung cancer; BRC, invasive ductal carcinoma of breast; PAC, pancreatic cancer; OVC, ovarian cancer; PRC, prostatic cancer; TPF, true positive fraction; FNF, false negative fraction; TNF, true negative fraction; FPF, false positive fraction; PPV, positive predictive value; NPV, negative predictive value; LR, likelihood ratio; DOR, diagnostic odds ratio
